# Aberrantly expressed miR-188-5p promotes gastric cancer metastasis by activating Wnt/β-catenin signaling

**DOI:** 10.1186/s12885-019-5731-0

**Published:** 2019-05-28

**Authors:** Yun Li, Xiaoli Yan, Jiajian Shi, Yun He, Jie Xu, Liying Lin, Wannan Chen, Xinjian Lin, Xu Lin

**Affiliations:** 1 Key Laboratory of Gastrointestinal Cancer (Fujian Medical University), Ministry of Education, Fuzhou, China; 2 Institute of Tissue Transplantation and Immunology and Department of Immunobiology, College of Life Science and Technology, Jinan University, Guangzhou, China; 3 Fujian Key Laboratory of Tumor Microbiology, Fujian Medical University, Fuzhou, China

**Keywords:** MiR-188-5p, Gastric cancer, Metastasis, Wnt/β-catenin signaling

## Abstract

**Background:**

Gastric cancer (GC) is one of the most common human cancers with the high rate of recurrence, metastasis and mortality. Aberrantly expressed microRNAs (miRNAs) are associated with invasion and metastasis in various human cancers. Recently, miR-188-5p has been indicated as an oncogene in GC since it promotes GC cell growth and metastasis. However, the underlying molecular mechanism remains to be fully defined.

**Methods:**

Using Significance Analysis of Microarrays (SAM) screening, we identified that miR-188-5p is associated with overall survival and lymph node metastasis in patients with GC. The functional impact of miR-188-5p on GC metastasis was validated using in vitro and in vivo assays. The regulatory function of miR-188-5p on Wnt/β-catenin signaling activation through directly targeting PTEN was proven using quantitative real-time PCR, western blot analysis, a dual-luciferase assay, a Transwell assay, and immunofluorescence. Immunohistochemical analyses further confirmed the clinical significance of miR-188-5p in GC.

**Results:**

MiR-188-5p diminishes tumor suppressor PTEN expression, and further increases phospho-Ser9 of GSK3β to activate Wnt/β-catenin signaling in GC. Consequently, miR-188-5p enhanced the migration and invasion of GC cells in vitro and tumor metastasis in vivo, whereas inhibition of miR-188-5p had the opposite effects. Moreover, miR-188-5p was negatively correlated with PTEN expression but positively correlated with nuclear β-catenin staining in GC samples.

**Conclusions:**

Our findings revealed a model of the miR-188-5p-PTEN-β-catenin axis in GC, which mediates the constitutive activation of Wnt/β-catenin signaling and promotes tumor metastasis, inferring that miR-188-5p is a potential therapeutic target to treat GC.

**Electronic supplementary material:**

The online version of this article (10.1186/s12885-019-5731-0) contains supplementary material, which is available to authorized users.

## Background

Gastric cancer (GC) is characteristic of poor prognosis and high mortality, making it the third most widely diagnosed malignancy worldwide and the cause of 12% of all cancer-related deaths each year [1]. Tumor metastasis is common and represents a major obstacle to improvements in GC patient survival [2]. Therefore, there is a urgent need to identify new prognostic markers and to develop novel therapeutic strategies to treat metastatic GC. The evolution of gastric tumorigenesis involves a series of sequential steps. Among these characteristic events of GC metastasis is hyperactivation of Wnt/β-catenin signaling. Activated Wnt/β-catenin signaling correlates with aggressiveness of GC with poor patient survival [3]. Moreover, a high level of β-catenin activity, stimulated by abnormal expression of Wnt-5a, functionally enhances GC cells’ migratory and invasive abilities [4]. Conversely, ablation of β-catenin resulted in suppression of metastasis in GC [5].

Wnt/β-catenin signaling is triggered upon binding of active Wnt ligands to their membrane receptors frizzled (FZD) [6] and LDL receptor-related protein 5 or 6 (LRP5/6) [7]. The ligand-receptor interaction can cause dissociation of β-catenin from the “destruction complex” comprising Axin, APC, CK1α, and GSK3β [8]. As a consequence, increased free cytosolic β-catenin translocates to the nucleus to induce the transcription of its downstream genes related to tumorigenesis. In the absence of Wnt ligand stimulation, cytosolic β-catenin is normally sequestrated in the “destruction complex” and Wnt/β-catenin signaling is inactivated [9, 10]. Additionally, crosstalk between Wnt/β-catenin signaling and other pathways such as PI3K/Akt pathway has been implicated in numerous cancers [11]. The tumor suppressor gene, phosphatase and tensin homolog (*PTEN*), is frequently inactivated in various tumor types. In addition, that PI3K/Akt suppression inhibits β-catenin activity is one of the major mechanisms by which PTEN exerts its suppressive effect on tumor progression [12, 13]. While signaling in Wnt/β-catenin pathway is deliberately controlled, its constitutive activation is frequently observed in many human cancer types. Therefore, revealing how these factors regulate the Wnt/β-catenin signaling pathway in cancers is of biological and clinical important for the future development of more efficacious therapeutic strategies.

MicroRNAs (miRNAs or miRs) are short non-coding RNAs that bind to 3′-untranslated regions (UTRs) of the mRNAs of target genes causing their post-transcriptional inhibition or degradation [14]. Besides their crucial roles in cellular differentiation and organism development, miRNAs are frequently misregulated in human cancer [15, 16]. Distinct miRNAs could act as key regulators of the Wnt/β-catenin signaling pathway in a variety of human malignancies including breast [17], liver [18] and colon cancer [19]. However, whether miRNA dysregulation could interfere with important Wnt/β-catenin signaling suppressors for aberrant activation of this signaling pathway in GC remains largely unknown.

In the present study, we identified miR-188-5p as an important miRNA that is closely correlated with GC metastasis and poor patient survival. Mechanistically, by inhibiting PTEN expression, miR-188-5p appears to function as the activator of the Wnt/β-catenin pathway leading to an increase in GC cell invasion in vitro and tumor metastasis in vivo. These results suggest that miR-188-5p contributes to promoting GC metastasis, and may represent a novel therapeutic target for the treatment of GC.

## Methods

### Cell culture

A total of seven human GC cell lines (MKN74, AGS, KATOIII, NUGC3, MGC803, MKN45, and HGC27) were purchased from the Type Culture Collection of the Chinese Academy of Sciences (Shanghai, China) and maintained in RPMI-1640 medium (Invitrogen, Carlsbad, CA, USA) except AGS in Ham’s F12 medium (Invitrogen). All media were supplemented with 10% (v/v) fetal bovine serum (Invitrogen).

### The Cancer genome atlas (TCGA) data analysis

miRNA-seq data from 378 stomach tumor samples with N stage information were downloaded from the TCGA stomach cancer data set portal (http://tcga-data.nci.nih.gov/tcga accessed on 2016/3/10; TCGA accession codes are listed in Additional file 1: Excel spreadsheet 1. Among these 378 stomach tumor samples, 307 samples also had overall survival (OS) data. Clinical information on the recruited patient specimens is shown in Additional file 2: Table S1. BRB-Array Tools [20] were used to perform the data analysis. The exclusion criteria for a given miRNA were as follows: less than 20% of the expression data showing at least a 1.5-fold change in either direction from the miRNA’s median value, or the percentage of data missing or filtered out exceeding 50%.

### Tissue specimens

GC samples and their corresponding adjacent non-tumor tissues were collected and histopathologically diagnosed at the Union Hospital of Fujian Medical University (Fuzhou, China) and relevant clinical information on the recruited patients is presented in Additional file 3: Table S2. These specimens are designated here as the FMU cohort. The patients’ informed consent and approvals from the Institutional Research Ethics Committee were obtained.

### Significance analysis of microarray data

Microarray data were analyzed using MeV4.9 (http://www-stat-class.stanford.edu/SAM/SAMServlet) with the SAM (significance analysis of microarrays) program (Stanford University, Stanford, CA, USA) [21] which identifies the genes most closely correlated with survival time or lymph node metastasis status with simultaneous permutation analysis for estimation of the false discovery rate (FDR).

### Gene set enrichment analysis (GSEA)

Global mRNA expression profiles on the TCGA stomach cancer specimens with the availability of miR-188-5p expression data were subjected to GSEA using GSEA 2.0.9 software (http://www.broadinstitute.org/gsea/) for identifying an association of miR-188-5p with metastasis-related and Wnt/β-catenin signaling pathways, using the methods as previously described [22, 23]. A numeric variable was assigned to miR-188-5p expression and a continuous-type cls file of the miR-188-5p profile was applied to phenotype the labels in GSEA. The metric for ranking genes in GSEA was set as ‘Pearson’ whereas the other parameters were set to their default values [24].

### Plasmids and transfection

A cDNA including the hsa-miR-188 precursor with 500 bp of flanking genomic sequence on each side was cloned into the retroviral transfer plasmid pMSCV-puro (Clontech, Mountain View, CA, USA). The open reading frame (ORF) of *PTEN* was inserted into the mammalian expression vector pcDNA 3.1 (Invitrogen). The 3′-UTR of *PTEN* was placed downstream of the luciferase gene in a pmirGLO dual-luciferase miRNA target expression vector (Promega, Madison, WI, USA). The primers used are listed in Additional file 4: Table S3. MiR-188-5p mimic, miR-188-5p inhibitor, and their control oligonucleotides were obtained from GenePharma (Shanghai, China). PTEN-TSB, a customized target site blocker (TSB) to block miR-188-5p binding to *PTEN*, and non-targeting control TSB LNA oligonucleotides were purchased from Exiqon (Vedbaek, Denmark). Transfection of plasmids or oligonucleotides was performed using X-tremeGENE reagent (Roche Diagnostics, GmbH, Mannheim, Germany) according to the manufacturer’s instructions.

### RNA extraction and quantitative real-time PCR

Total miRNA isolation and cDNA synthesis were performed using commercial kits according to the manufacturer’s instructions. miRNA expression was quantified with qPCR using miRNA-specific primers in a real-time PCR system as previously described [25]. Additional file 5: Table S4 shows the sequences of the primers used.

### Western blot analysis and immunofluorescent assays

Western blotting and immunofluorescent assays were performed according to corresponding standard methods [25]. The specific antibodies used were listed as follows: PTEN (Cell Signaling Technology (CST), 1:1000 dilution), GSK3β (CST, 1:800 dilution), Akt (CST, 1:800 dilution), phospho (p) Akt (Ser473) (CST, 1:500 dilution), pGSK3β (Ser9) (CST, 1:500 dilution), GAPDH (CST, 1:5000 dilution), β-catenin (Abcam, 1:50 dilution), and Alexa Fluor 594-conjugated goat anti-rabbit secondary antibody (1:200, 2 mg/mL, Invitrogen). Fluorescence images were captured by a laser scanning confocal microscope (Zeiss, Germany).

### Cell proliferation assay

Cells were seeded at a density of 5 × 10^3^ cells per well in 96-well plates and cultured for 24, 48, 72, 96, or 120 h. Cell Counting Kit-8 (CCK-8; Dojindo, Kuma-moto, Japan) was used to evaluate the proliferative capacity of the cells. The absorbance was then measured at the wave length of 450 nm using a microplate reader (Bio-Tek, Winooski, VT, USA).

### Wound-healing assay

Cells were grown to nearly 100% confluence on 6-well cell culture dishes and a same size scratch was made through the cell monolayer using a pipette tip. After thorough washing with PBS, fresh culture medium was replenished and the wound closure was photographed at 0, 24 and 48 h to monitor the migration of cells into the wounded area.

### Cell migration and invasion assay

3 × 10^4^ cells were placed onto the upper chamber of Transwell insert (BD bioscience, San Jose, CA, USA) without or with Matrigel and incubated at 37 °C for 22 h followed by removal of cells inside the upper chamber with cotton swabs. The insert was then fixed in 20% methanol, stained with 0.1% crystal violet and counted under Qimaging micropublisher 5.0 RTV microscope (Olympus, Tokyo, Japan).

### Dual-luciferase reporter assay

3.5 × 10^4^ cells were seeded and settled in 24-well plates for 24 h. Indicated plasmids (100 ng each) plus 1 ng of pRL-TK plasmid (Promega) were transfected into cells. For measurements of β-catenin transcriptional activity, the reporter plasmid containing the DNA binding site of wild-type (CCTTTGATC; TOP-flash) or mutated (CCTTTGGCC; FOP-flash) TCF/LEF (Addgene, Cambridge, MA, USA) were used. Luciferase and Renilla signals were measured 48 h after transfection using the Dual-Luciferase Reporter Assay Kit (Promega) according to a protocol provided by the manufacturer.

### Tumor xenografts in vivo

3-million MGC803 cells or 4-million AGS cells stably expressing firefly luciferase and miR-188-5p were injected intravenously via the tail vein of BALB/c nude mice. Fifty days after inoculation, the mice were euthanized by carbon dioxide inhalation followed by cervical dislocation and the lungs were removed and examined for any evidence of metastasis by a dissecting microscopy and histopathologic analysis. IVIS Spectrum Imaging System (Caliper Life Sciences, Hopkinton, MA, USA) was employed for a whole-mouse bioluminescence imaging. Living Image 4.2 software (Caliper Life Sciences) was used for image calibration and visualization. The animal study was approved by the Institutional Animal Care and Use Committee of Fujian Medical University.

### Immunohistochemistry

Immunohistochemistry was performed according to the standard streptavidin-biotin- peroxidase complex method. After sections were stained using anti-PTEN (CST, 1:100 dilution) and anti-β-catenin (CST, 1:100 dilution), images were captured using the AxioVision Rel.4.6 computerized image analysis system (Carl Zeiss). The degree of immunostaining of indicated proteins was evaluated and scored as previously described [25].

### Statistical analysis

All statistical analyses were carried out using the SPSS 20.0 statistical software package except for the microarray data. Power analysis was performed to determine sample size in order to achieve a minimum effect size of 0.5 with a *P* value < 0.05. The patients were classified into two groups of a high (> the median) or low (≤ the median) miR-188-5p expression. Overall survival curves were plotted by the Kaplan-Meier method with the log-rank test applied to determine a significance. Cox regression was used for analysis of univariate and multivariate survival. The relationship between the expression levels of miR-188-5p and its target genes was analyzed by *χ*^2^-test or Fisher’s exact test. Student’s *t*-test was used to determine differences between groups. All data were represented as mean ± SD from 3 independent experiments. *P* < 0.05 was considered significant.

## Results

### miR-188-5p correlates with GC patient survival and lymph node metastasis

To examine whether miRNAs were associated with GC patient survival and lymph node metastasis status, we analyzed the miRNA expression profiles in patients with stomach adenocarcinoma (STAD) using the stomach cancer data set of TCGA. From censored survival analysis of the Significance Analysis of Microarrays (SAM) in MeV4.9, we identified 13 distinctly expressed miRNAs that were closely correlated with OS or lymph node metastasis status, corresponding to a median expected FDR of 10% (Table 1). Among the 13 miRNAs identified, miR-221-3p and miR-223-3p have been reported of clinical significance in GC [26, 27]. Intriguingly, miR-188-5p appeared to be the only miRNA significantly correlated with both OS and lymph node metastasis status. Moreover, miR-188-5p expression level was much higher than miR-188-3p in the TCGA miRNAHiSeq expression array (Additional file 6: Figure S1A). These findings indicated that miR-188-5p might be potentially more important than miR-188-3p in GC progression.

**Table 1:** Significance analysis of microarrays (SAM) using the TCGA Stomach Adenocarcinoma (STAD) data set identified microRNAs associated with patients’ OS or lymph node metastasis

microRNAs	*q*-value(%)
High expression correlated with shorter OS	
hsa-miR-221-3p	0.00
hsa-miR-188-5p	0.00
hsa-miR-223-3p	0.00
Low expression correlated with shorter OS	
hsa-miR-145-5p	0.00
hsa-miR-29a-3p	0.00
hsa-miR-200b-3p	0.00
hsa-miR-194-5p	3.47
High expression correlated with positive-lymph node metastasis	
hsa-miR-125a-5p	0.00
hsa-miR-500a-3p	0.00
hsa-miR-188-5p	0.00
Low expression correlated with positive-lymph node metastasis	
hsa-miR-211-5p	0.00
hsa-miR-147b	0.00
hsa-let-7b-5p	0.00
hsa-miR-150-5p	0.00

*OS* overall survival

**Fig. 1:**
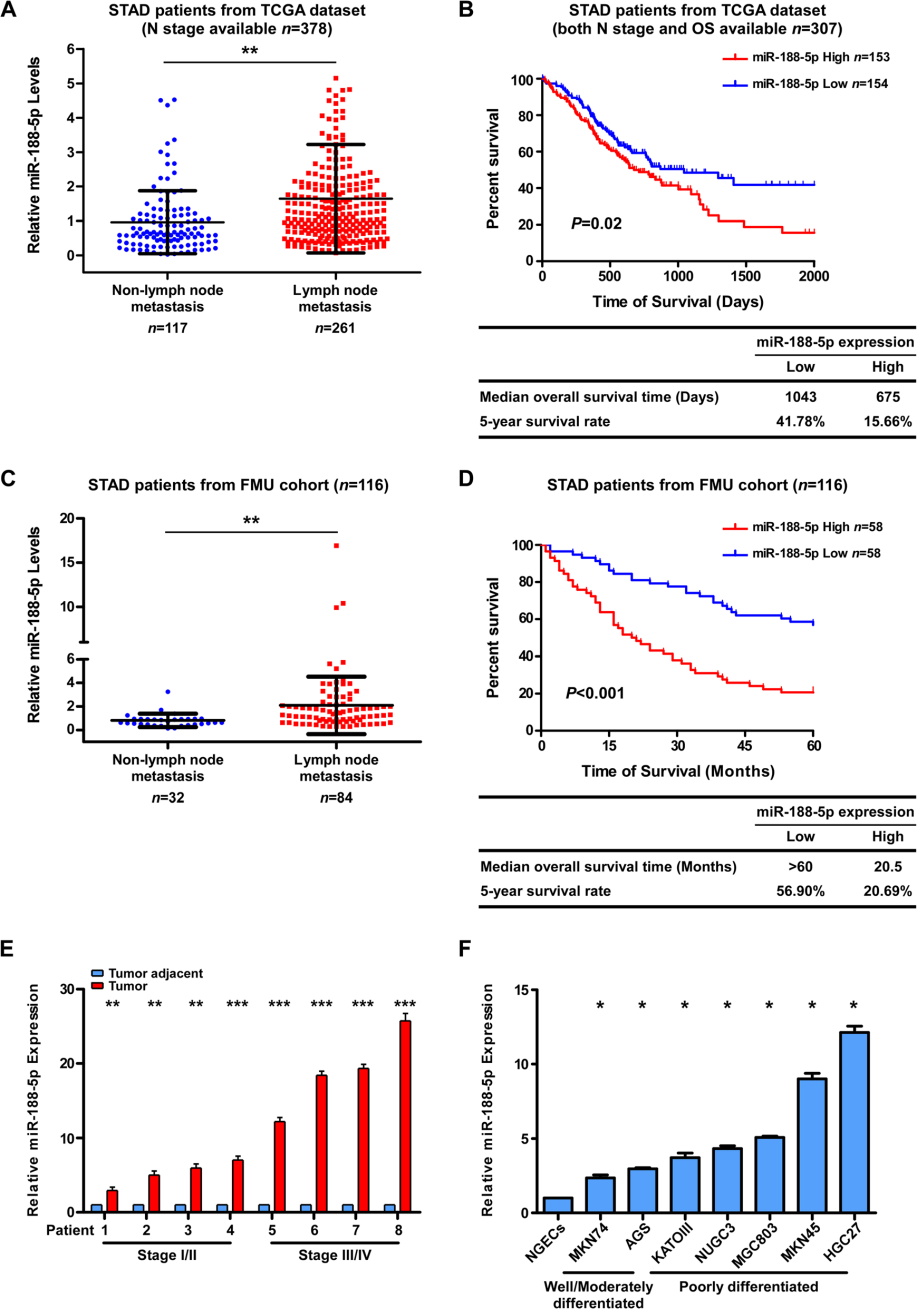
Upregulation of miR-188-5p in GC is correlated with poor prognosis and lymph node metastasis. **a** Expression levels of miR-188-5p were analyzed in patients with (*n* = 261) and without metastasis (*n* = 117), whom N stage information was available in the TCGA stomach cancer dataset. **b** Kaplan–Meier analysis of the OS of patients with STAD for whom both N stage and OS information was available in the TCGA stomach cancer dataset. The patients were stratified by high (greater than the median, *n* = 153) versus low (less than or equal to the median, *n* = 154) expression of miR-188-5p. **c** qPCR analysis of the expression level of miR188-5p in tumors with (*n* = 84) and without metastatic relapse (*n* = 32) in the FMU cohort. **d** Kaplan–Meier analysis of the correlation between the miR-188-5p level and the 5-year OS of 116 patients with STAD in the FMU cohort. The patients were stratified by high (greater than the median, *n* = 58) versus low (less than or equal to the median, *n* = 58) expression of miR-188-5p. **e** Relative expression of miR-188-5p in eight pairs of GC tumor tissues and their corresponding adjacent non-cancerous tissues. **f** qPCR analysis of miR-188-5p expression in gastric epithelial cells, including primary NGECs (normal gastric epithelial cells) and a panel of seven human GC cell lines. Relative expression levels were normalized by U6 expression. Error bars represent mean ± SD from three independent experiments.***, *P* < 0.001; **, *P* < 0.01; *, *P* < 0.05. FMU, Fujian Medical University; GC gastric cancer; NGECs, normal gastric epithelial cells; OS, overall survival; qPCR; quantitative real-time PCR; STAD, stomach adenocarcinoma; TCGA, The Cancer Genome Atlas

More specifically, for those STAD patients in the TCGA data set with N stage data available, the expression of miR-188-5p was markedly higher in stage N1–N3, but significantly lower in stage N0 STAD samples (unpaired Student’s *t*-test, *P* < 0.01, Fig. 1a). MiR-188-5p was highly upregulated in lymph node metastatic GC specimens as compared with non-lymph node metastatic samples. Furthermore, as shown in Fig. 1b, the patients with a higher miR-188-5p expression (> median) had a significantly shorter OS than those expressing a lower level of miR-188-5p (≤ median). Similar results were seen in the FMU cohort (Fig. 1c and d). Moreover, an additional analysis of miR-188-5p expression between M0 and M1 patients in TCGA STAD cohort also revealed that miR-188-5p was significantly upregulated in M1 patients as compared with M0 patients (unpaired Student’s t-test, *P* = 0.0332; Additional file 6: Figure S1B), suggesting that miR-188-5p level is correlated with tumor distant metastasis in GC patients.

We then evaluated the expression level of miR-188-5p in tumor specimens and GC cell lines. Figure 1e showed that expression of miR-188-5p was increased in eight randomly selected GC tumor specimens as compared with the paired adjacent non-tumor tissues. The same was true for seven GC cell lines relative to normal gastric epithelial cells (NGECs) (Fig. 1f). To further confirm this, we also analyzed miR-188-5p expression levels in TCGA STAD cohort that demonstrated miR-188-5p was significantly up-regulated in gastric cancers compared to normal tissues (*P* < 0.0001; Additional file 6: Figure S1C). Similarly, analysis of paired adjacent normal and GC tissues revealed that the miR-188-5p level was more than 5-fold increased in GC samples (Additional file 6: Figure S1D and E). Collectively, these data indicated that miR-188-5p was markedly upregulated in GC.

### miR-188-5p promotes migration, invasion, and metastasis of GC cells

**Fig. 2:**
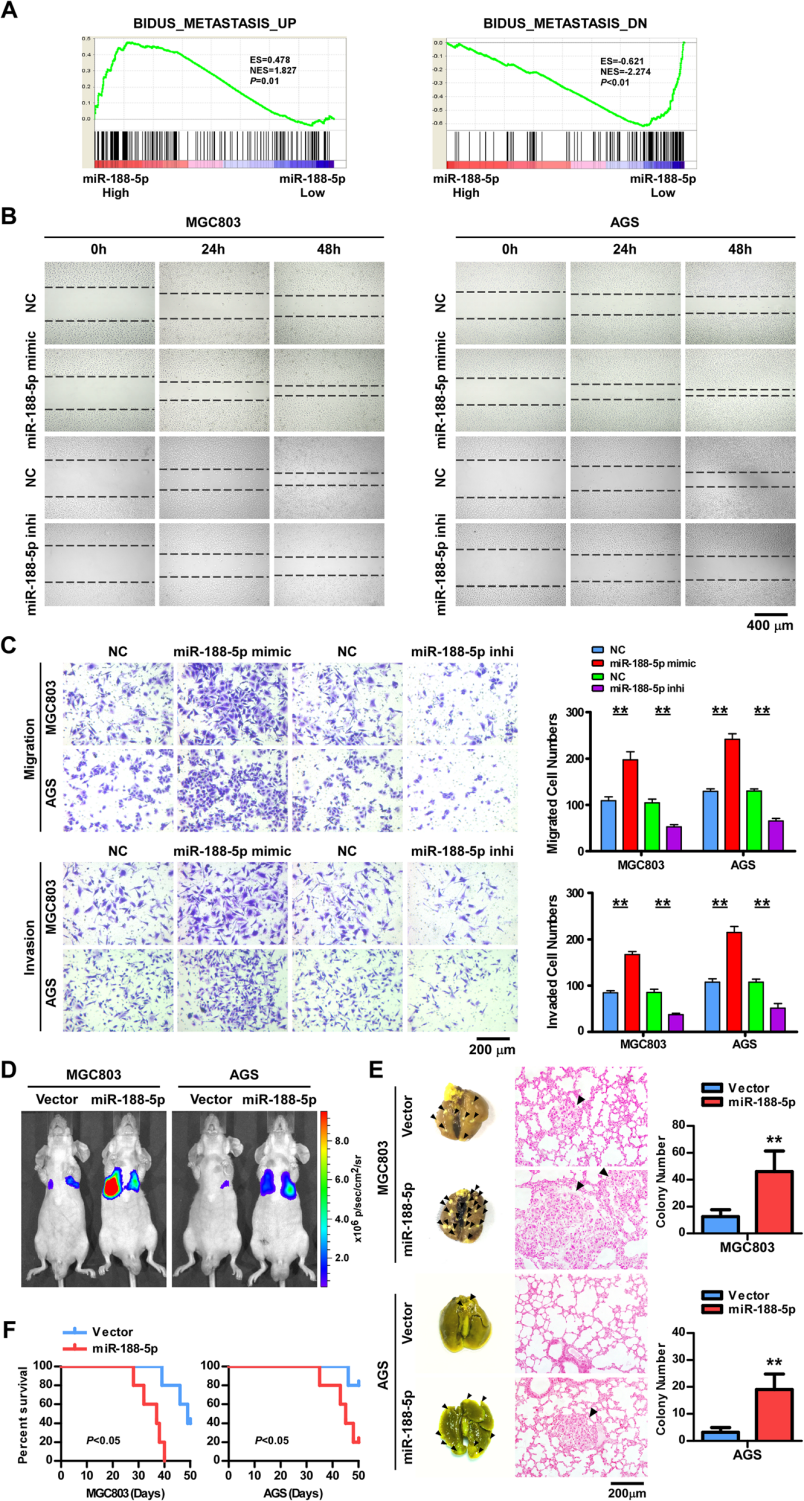
miR-188-5p promotes migration, invasion, and metastasis of GC cells. **a** GSEA plot showing miR-188-5p expression in association with metastasis-related gene signatures. The miR-188-5p level was positively correlated with activated metastasis-related gene signatures and inversely correlated with suppressed metastasis-related gene signatures in the TCGA stomach cancer dataset. **b** A wound-healing assay was performed with the indicated cells; Scale bar, 400 μm. **c** Representative images and quantification of Transwell assays in the indicated cells; Scale bar, 200 μm. **d** BALB/c nude mice injected intravenously via the caudal vein with indicated cells were monitored with a luciferase live-imaging system at indicated time points. Heat map scale bar represents photon emission. **e** Representative bright-field imaging of the lungs on 50 days of mice (left); H&E staining of lung metastases formed by the indicated cells (middle) and number of visible surface metastatic lesions in mice (*n* = 5 per group) injected intravenously with indicated cells (right), Scale bar, 200 μm. Error bars represent mean ± SD from three independent experiments. **f** Survival curves of mice injected intravenously with indicated cells. Error bars represent mean ± SD (*n* = 5 per group). **, *P* < 0.01. GC gastric cancer; GSEA, gene set enrichment analysis; H&E, hematoxylin and eosin; NC, normal control; TCGA, The Cancer Genome Atlas

To elucidate the biological function of miR-188-5p in GC carcinogenesis, we transfected MGC803 (poorly differentiated) and AGS (moderately differentiated) cells with the miR-188-5p mimic or inhibitor. Overexpression of miR-188-5p did not alter the proliferation of MGC803 or AGS cells, as determined by CCK-8 assays (Additional file 7: Figure S2). However, GSEA of the TCGA STAD cohort showed that a higher level of miR-188-5p was positively correlated with the gene signatures upregulated in metastatic cancer cells but inversely correlated with the gene signatures downregulated in metastasis cells (Fig. 2a) [22]. Furthermore, the migratory and invasive abilities of MGC803 and AGS cells were markedly enhanced by the miR-188-5p mimic but suppressed by the inhibitor, as determined by wound-healing/scratch assay (Fig. 2b) and Boyden chamber invasion assay (Fig. 2c).

The effect of miR-188-5p on metastatic potential in vivo was evaluated by generating the MGC803/miR-188-5p or AGS/miR-188-5p cells expressing luciferase and then injecting 2 × 10^6^ cells into the caudal vain of BALB/c nude mice. Additional file 8: Figure S3 showed that miR-188-5p expression was markedly increased in miR-188-5p- overexpressing GC cells as compared with the control cells. Whole-mouse bioluminescence imaging was employed to serially monitor the appearance of metastases in living mice and the metastatic nodules on the lung surface of sacrificed mice were counted and detected by H&E staining. Mice injected with MGC803 or AGS/miR-188-5p cells generated a significantly larger number of nodules in their lungs than those injected with the control cells (Fig. 2d and e). Notably, all the five mice in the group injected with MGC803/miR-188-5p cells died before 40 days after implantation while only three mice in the control group died by day 50 after inoculation (Fig. 2f, left). Likewise, AGS/miR-188-5p injected mice survived for a shorter time than those injected with the control cells (Fig. 2f, right). Altogether, these results indicate that miR-188-5p functions to promote GC cells migration, invasion, and metastasis.

### miR-188-5p directly targets tumor suppressor PTEN

**Fig. 3:**
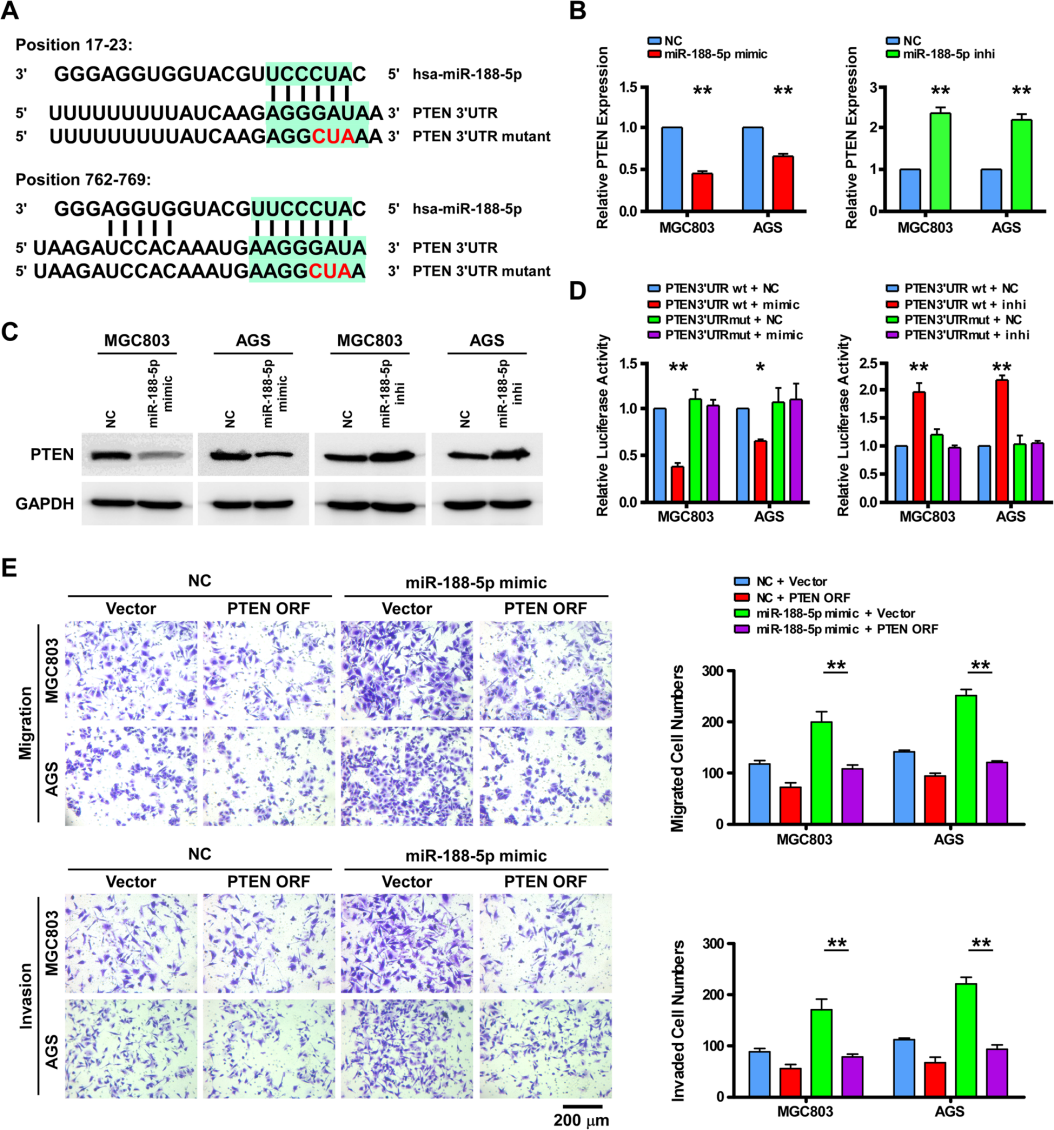
miR-188-5p directly targets tumor suppressor PTEN. **a** Predicted binding sites of miR-188-5p in the wild-type 3′-UTR of PTEN. Mutations in the 3′-UTR are highlighted in red. **b** and **c** qPCR and western blotting analyses of PTEN expression in the indicated cells. **d** Luciferase activity of reporters with wild-type or mutant 3′-UTRs of PTEN in the indicated cells co-transfected with the indicated oligonucleotides. **e** The effect of transducing the ORF (without the 3′-UTR) of PTEN in a Transwell assay in the indicated control miRNA (NC)- or miR-188-transfected cells; Scale bar, 200 μm. Error bars represent mean ± SD from three independent experiments. **, *P* < 0.01; *, *P* < 0.05. NC, normal control; ORF, open reading frame; qPCR; quantitative real-time PCR; PTEN, phosphatase and tensin homolog; UTR, untranslated region

To explore the underlying mechanism by which miR-188-5p had the robust effect on GC metastasis, we first attempted to narrow down miR-188-5p target genes using TargetScan v6.2. The tumor suppressor gene, *PTEN*, was found to be suppressed by miR-188-5p (Fig. 3a). qPCR and western blot analysis found that both mRNA and protein levels of PTEN were drastically reduced in cells overexpressing miR-188-5p, but were elevated in cells transfected with the miR-188-5p-inhibitor (Fig. 3b and c). Subsequently, the 3′-UTR of *PTEN* was cloned into a luciferase reporter plasmid to evaluate whether miR-188-5p could bind to the *PTEN* 3′-UTR leading to inhibition of luciferase reporter activity. As expected, a decrease in luciferase reporter activity in response to miR-188-5p expression was observed in MGC803 and AGS cells whereas miR-188-5p overexpression did not have inhibitory effects when the predicted miR-188-5p target/binding sites in the PTEN 3′-UTR region were mutated (Fig. 3d). Therefore, these results strongly confirmed that miR-188-5p directly interacts with *PTEN* as a target.

To investigate the functional significance of miRNA-mediated suppression of *PTEN* expression in the induction of cellular metastatic ability, we restored PTEN expression by transfecting a PTEN ectopic expression plasmid. As illustrated in Fig. 3e, PTEN overexpression inhibited the migratory and invasive capabilities of both the NC and miR-188-5p overexpressing GC cells in Transwell assays. Taken together, our data implicate that miR-188-5p promotes GC cell migration and invasion by suppressing *PTEN*.

### miR-188-5p promotes Wnt/β-catenin signaling

**Fig. 4:**
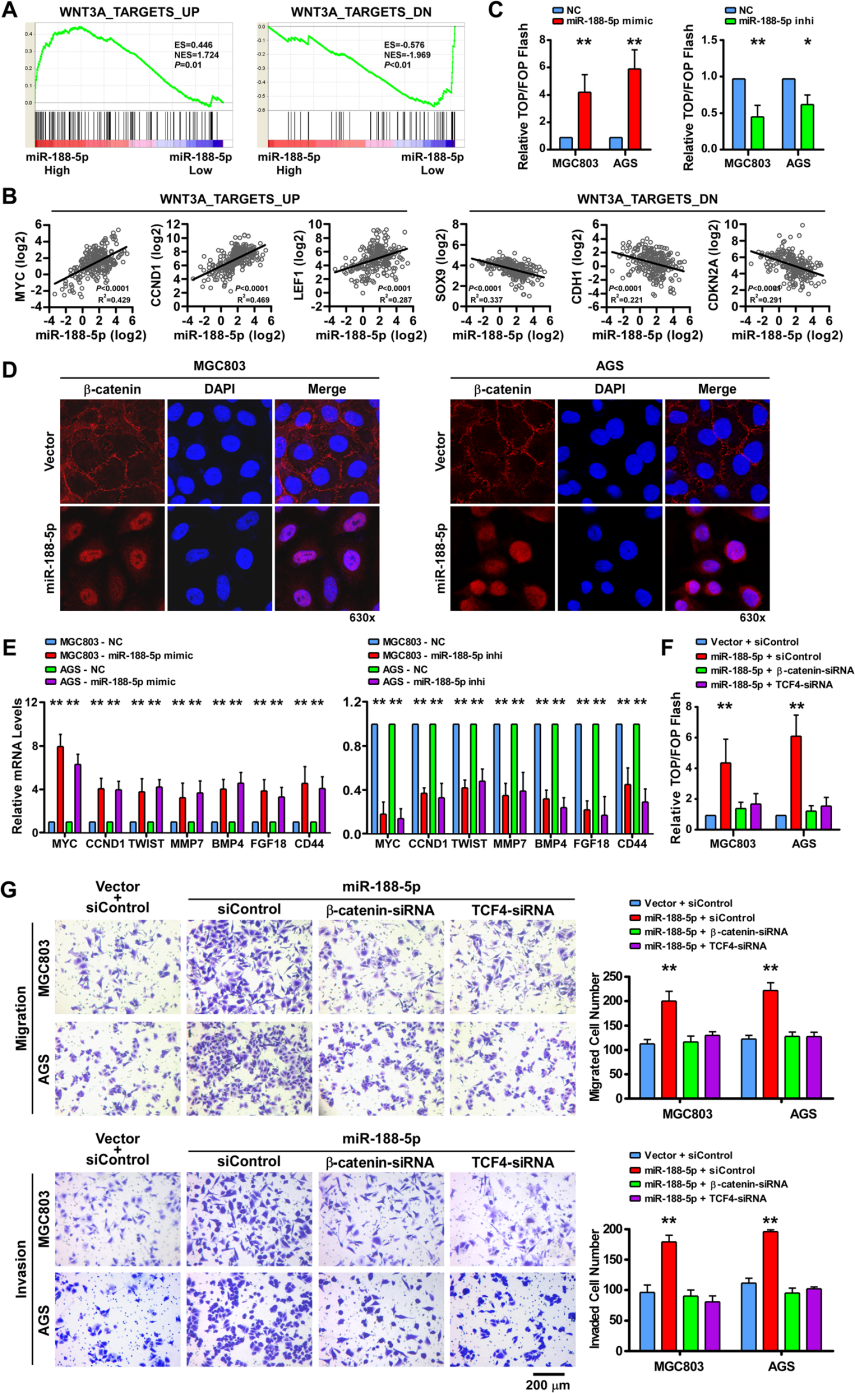
miR-188-5p promotes Wnt/β-catenin signaling. **a** GSEA plot showing that miR-188-5p expression was positively correlated with Wnt-activated gene signatures and inversely correlated with Wnt-suppressed gene signatures in the TCGA stomach cancer dataset. **b** A linear regression analysis on the expression of Wnt pathway genes (the representative 3 upregulated and 3 downregulated Wnt target genes) versus miR-188-5p in the TCGA stomach cancer dataset. **c** The indicated cells were transfected with TOP or FOP reporter and *Renilla* pRL-TK plasmids and subjected to dual-luciferase assays 48 h after transfection. The detected reporter activity was normalized to the *Renilla* activity. **d** Subcellular β-catenin localization in the indicated cells was assessed by immunofluorescence staining; 630×. **e** qPCR analysis of the expression of the established downstream targets for the Wnt/β-catenin pathway, including *MYC*, *CCND1*, *TWIST*, *MMP7*, *BMP4*, *FGF18*, and *CD44*, in the indicated cells. **f** Assay of TOP/FOP luciferase activity in the indicated cells. **g** Representative images of a Transwell assay in the indicated cells (left) and quantification of Transwell assay in the indicated cells (right). Scale bar, 200 μm. Error bars represent mean ± SD from three independent experiments.**, *P* < 0.01; *, *P* < 0.05. GSEA, gene set enrichment analysis; NC, normal control; qPCR; quantitative real-time PCR; siRNA, short interfering RNA; TCGA, The Cancer Genome Atlas

Since the Wnt/β-catenin signaling pathway plays a crucial role in cancer metastasis we next examined whether miR-188-5p could impact Wnt/β-catenin signaling activity. The results from GSEA analysis using the TCGA stomach cancer data set revealed that the miR-188-5p level was in a positive correlation with Wnt-activated gene signatures but an inverse correlation with Wnt-suppressed gene signatures [23] (Fig. 4a and b), indicating that miR-188-5p might participate in Wnt/β-catenin signaling activation. To address this issue, Top/Fop Flash reporter assays were used to evaluate the effects of miR-188-5p on the β-catenin transcriptional activity in GC Fig. 4c showed that ectopic expression of miR-188-5p in MGC803 and AGS cells could significantly increase the β-catenin transcriptional activity whereas the miR-188-5p inhibitor demonstrated the opposite effects. Fluorescent microscopy was then utilized for detection of β-catenin intracellular localization. The results demonstrated that miR-188-5p overexpression in GC cells significantly enhanced β-catenin nuclear accumulation (Fig. 4d). We next examined the effects of miR-188-5p on the mRNA levels of the seven well-known effector genes of the Wnt/β-catenin signaling. Expectedly, miR-188-5p markedly enhanced the expression of the target genes while the miR-188-5p inhibitor reduced the expression (Fig. 4e).

Furthermore, we explored the role of Wnt/β-catenin activation in miR-188-induced metastasis. As shown in Fig. 4f and g, knockdown of β-catenin or TCF4 inhibited β-catenin transcriptional activity and substantially reversed the migratory and invasive ability of miR-188-5p-overexpressing GC cells, suggesting that miR-188-5p overexpression promote GC cells metastasis by activating Wnt/β-catenin signaling.

### miR-188-5p activates Wnt/β-catenin signaling via PTEN-Akt-GSK3β cascades

**Fig. 5:**
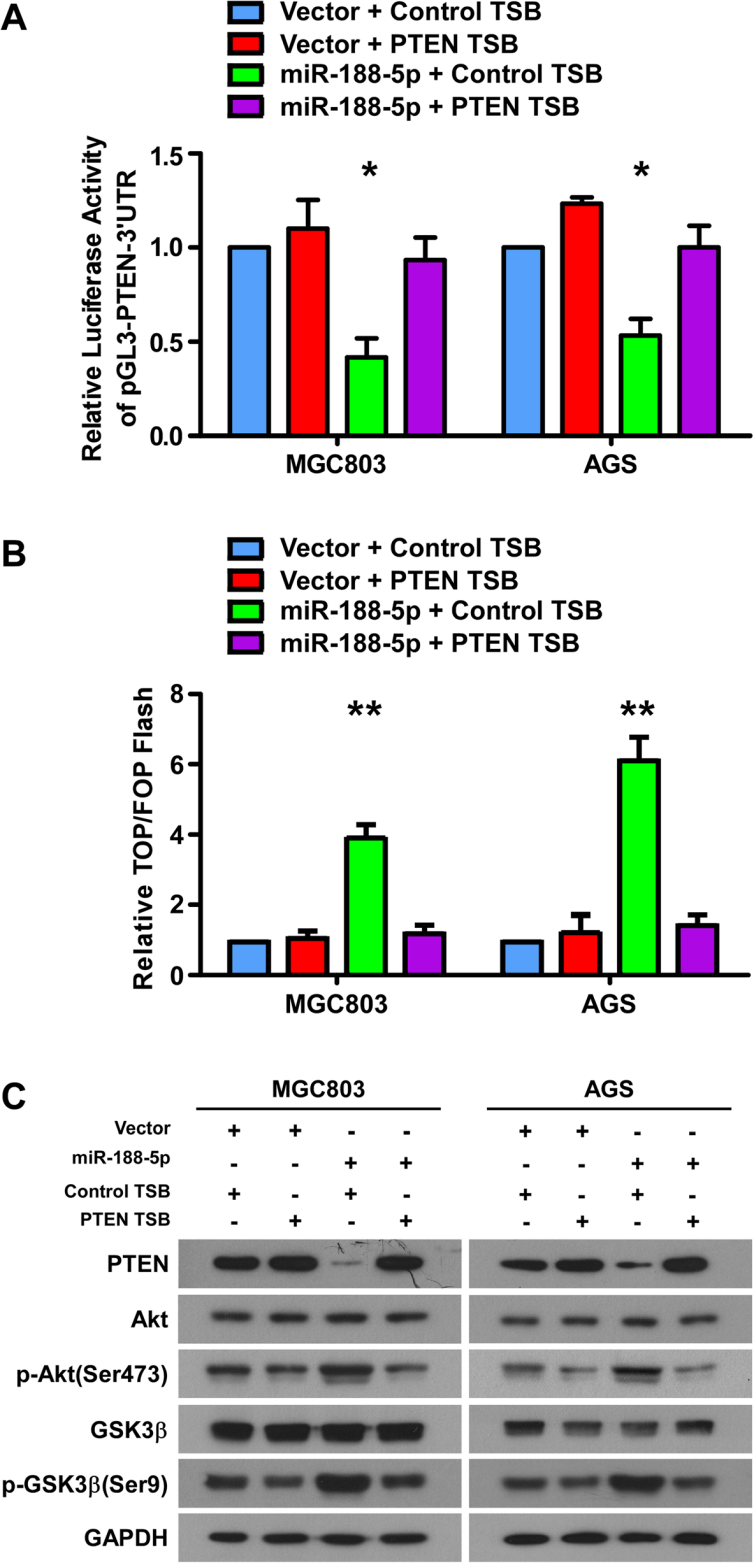
miR-188-5p activates Wnt/β-catenin signaling through PTEN-Akt-GSK3β cascades. **a** Luciferase assay of the 3′ UTR activity of PTEN in the indicated cells transfected with the indicated TSB. **b** Assay of TOP/FOP luciferase activity in the indicated cells. **c** Western blotting analyses of PTEN, Akt, p-Akt (Ser473), GSK3β and p-GSK3β (Ser9) expression in the indicated cells. Error bars represent mean ± SD from three independent experiments.**, *P* < 0.01; *, *P* < 0.05. NC, normal control; qPCR; quantitative real-time PCR; PTEN, phosphatase and tensin homolog; TSB, target site blocker; UTR, untranslated region

To determine the role of PTEN in miR-188-5p mediated Wnt/β-catenin signaling, we introduce PTEN-TSB to specifically inhibit the binding of miR-188-5p to PTEN mRNA in the control vector transfected and miR-188-5p overexpressing GC cells. The results showed that PTEN-TSB not only abrogated the inhibitory effects of miR-188-5p on the 3′ UTR of *PTEN* (Fig. 5a) but also reversed the effect of miR-188-5p on promoting β-catenin transcriptional activity (Fig. 5b). These results strongly supported the notion that miR-188-5p could specifically inhibit *PTEN* and activates Wnt/β-catenin signaling.

Crosstalk between the PI3K/Akt and Wnt/β-catenin pathway has been established in several cancer types including GC [28–31]. In this crosstalk, PTEN phosphorylates and inactivates GSK3β via Akt, which results in β-catenin accumulation and shuttling to the nucleus, thereby increasing target gene transcription [32]. Subsequently, we assessed the status of the PI3k/Akt pathway in the context of miR-188-5p with PTEN TSB by examining the phosphorylation status of Akt and GSK3β. As shown in Fig. 5c, upregulation of miR-188-5p in MGC803 (left) and AGS (right) cells markedly decreased PTEN expression and increasing of phosphorylation of Akt (Ser473) and GSK3β (Ser9) compared with vector cells (lane 1 and 3). However, PTEN TSB could almost entirely reverse the activation of PI3k/Akt pathway in GC cells initially enhanced by miR-188-5p (lane 3 and 4). These results indicate that miR-188-5p enhances Wnt/β-catenin activation through the PTEN-Akt- GSK3β signaling cascade.

### Clinical relevance of miR-188-5p and β-catenin activation in GC

**Fig. 6:**
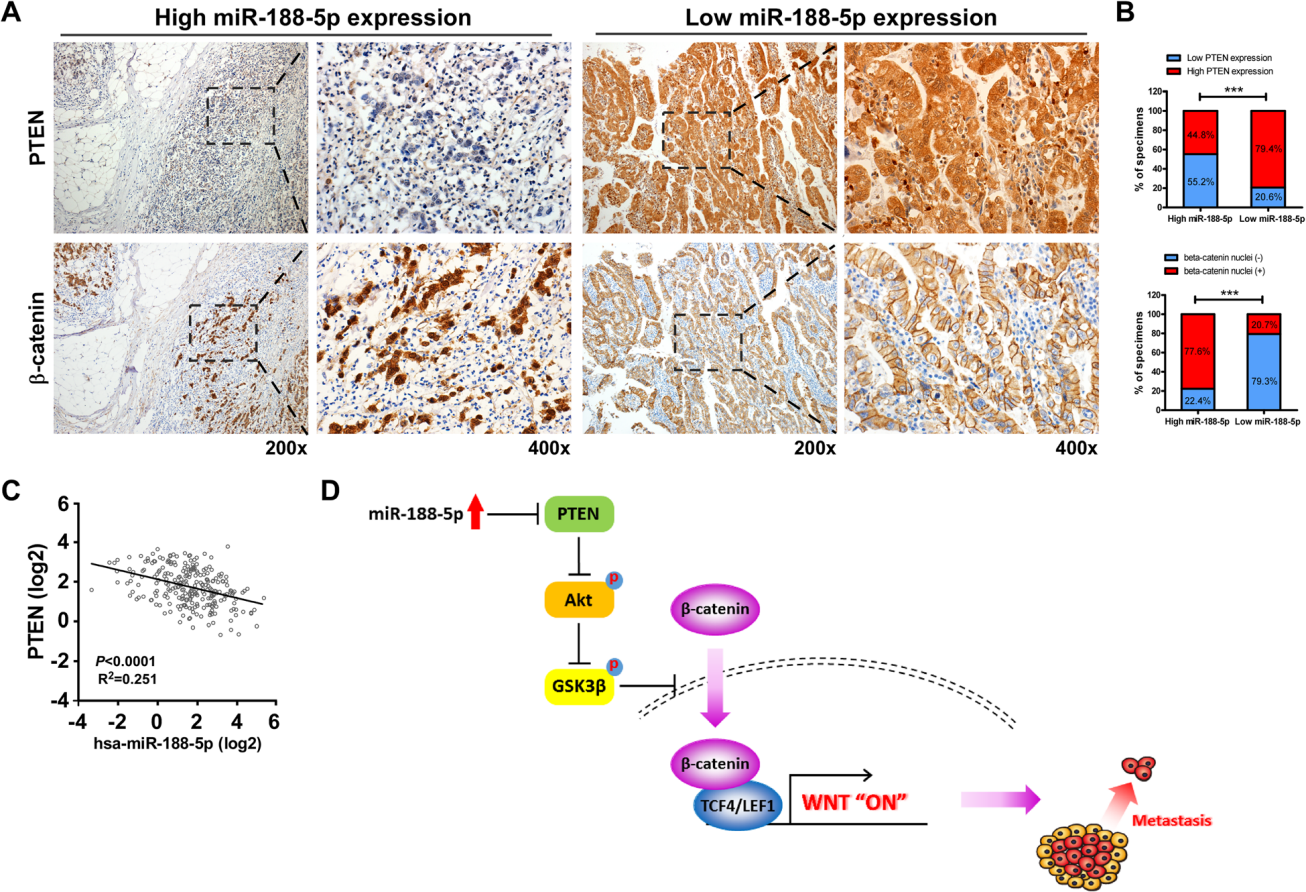
Clinical relevance of miR-188-5p expression in human GC tissue. **a** Immunohistochemical analysis of PTEN and β-catenin expression in serial sections of GC tumor specimens; 200×, 400×. **b** Percentage of specimens showing low or high miR-188-5p expression in relation to the expression levels of PTEN and nuclear β-catenin. ***, *P* < 0.001. **c** A linear regression analysis on the expression of PTEN versus miR-188-5p in the TCGA stomach cancer dataset. **d** Illustration of Wnt activation by miR-188-5p through suppression of PTEN. GC, gastric cancer; PTEN, phosphatase and tensin homolog

Finally, we evaluated whether miR-188-5p overexpression and its mediated activation of Wnt/β-catenin were of clinical relevance in GC. Figure 6a and b showed that there was an inverse correlation between the expression of miR-188-5p and the expression of PTEN in the FMU cohort of 116 GC clinical specimens. 55.2% of specimens with high miR-188-5p expression (32/58) showed low PTEN expression whereas 79.4% of specimens with low miR-188-5p expression (46/58) displayed high PTEN expression (χ^2^-test, *P* < 0.001). In particular, tumor specimens that expressed high levels of miR-188-5p also showed a more accumulation of nuclear β-catenin (based on immunohistochemistry) as compared with those expressing lower levels of miR-188-5p (77.6% vs. 20.7%). Consistently, the negative correlation between the expression of miR-188-5p and PTEN was also observed in the TCGA STAD cohort (Fig. 6c). Our data confirmed that miR-188-5p is negatively correlated with PTEN expression and positively correlated with nuclear β-catenin level.

## Discussion

Abnormal expression of miRNAs correlates with the tumorigenesis and progression of many human malignacies [14–16]. In the present study, the miRNA expression profiles of patients with STAD in the stomach cancer data set of TCGA were analyzed, and miR-188-5p was identified as the sole miRNA significantly correlated with both OS and lymph node metastasis status. This observation was further confirmed in a set of 378 patients with STAD in the TCGA data and FMU cohort, which showed that miR-188-5p was highly upregulated in lymph node metastatic GC specimens compared with its level in non-lymph node metastatic samples. Furthermore, miR-188-5p was also significantly upregulated in M1 patients as compared with M0 patients in TCGA STAD cohort. These data suggested that miR-155-5p expression level is positively correlated with GC metastasis.

miR-188-5p was reported to function as a tumor suppressor in several cancers including non-small-cell lung cancer, glioma, hepatocellular carcinoma and prostate cancer [33–36]. In contrast, miR-188-5p is also shown to be an oncogenic miRNA that promotes prostate cancer cell proliferation and invasion, and take parts in aggressive progression and poor prognosis in patients with prostate cancer via inhibiting its target gene, ubiquitin-conjugating enzyme E2I (UBE2I) [37]. In concert with this, in the present study we demonstrated that upregulation of miR-188-5p is involved in GC metastasis and poor patient survival. It is noteworthy that in a recent study, Peng et al. reported that miR-188-5p exerts tumor suppressor function in GC [38], which appears to disagree with our findings. The reasons for this discrepancy might be explained below. The TCGA data set and our FMU cohort used in the statistical analysis contained 378 and 116 GC samples respectively, which is greater than the sample number in Peng’s clinical cohort (*n* = 51). Using those much larger cohorts, we demonstrated that high miR-188-5p expression was significantly associated with poor survival of the GC patient and lymph node metastasis. In addition, it may also be notable that clinical information regarding the enrolled patients, pathological features and clinical stages was not available in Peng’s paper for an invitation of the comparison.

Since chromosomal amplification/deletion or epigenetic modification changes are important upstream mechanisms of abnormal miRNA expression, we went further to analyze copy-number alterations (CNAs) and DNA methylation profiles from TCGA STAD data set. We found that both the copy number and DNA methylation level at miR188 locus were barely altered in STAD samples with the frequency of alteration being less than 1%. In addition, we also employed the tool of Evolutionary Conserved Regions (ECR) Browser in attempt to identify distant gene regulatory elements and to predict transcription factor binding sites of miR188. We found that several signal transducer and activator of transcription (STAT) binding sites were enriched in the region of approximately 800 bp upstream of miR188 locus. Functional experimental validations to confirm a binding relationship and regulatory effects are needed in the future.

To examine the influence of miR-188-5p on the development and progression of GC, miR-188-5p was overexpressed or inhibited in GC cell lines AGS and MGC803. The results revealed that the migratory and invasive abilities of MGC803 and AGS cells were markedly enhanced by the miR-188-5p mimic, but were suppressed by the inhibitor. Consistently, an in vivo metastasis experiment also confirmed that miR-188-5p significantly increased the pulmonary colonization of metastatic tumors. These data pinpoint a crucial role of miR-188-5p that plays as a tumor promoter in GC cell invasion and metastasis.

MiRNAs can negatively regulate their target genes via binding to the 3′-UTR of their mRNA to induce cleavage of the mRNAs or inhibit their translation [14]. It has reported that miR-188-5p plays distinct roles in different type of cancers by directly targeting multiple genes in a variety of scenarios as such MMP-2/13, MAP3K3, ZFP91, UBE2I, β-catenin, FGF5, LAPTM4B, Cyclins and CDKs in non-small-cell lung cancer, gastric cancer, glioma, hepatocellular carcinoma, prostate cancer or nasopharyngeal carcinoma [33–40]. However, the precise role of miR-188-5p and the underlying molecular mechanism in gastric cancer remains to be defined. In the present study, prediction of the candidate target genes for miR-188-5p was made using bioinformatic methods combined with luciferase reporter assays, qPCR, and western blot analysis. We showed that miR-188-5p could bind directly to the 3′-UTR of *PTEN* and miR-188-5p overexpression downregulated PTEN expression at both the mRNA and protein levels. Moreover, restored expression of PTEN suppressed the migratory and invasive capabilities of both the NC and miR-188-5p overexpressing GC cells. Intriguingly, in a recent study of diabetic kidney disease (DKD) miR-188-5p was found to enhance renal tubular epithelial-mesenchymal transition (EMT) by a direct interaction with the PTEN 3′-untranslated region to suppress PTEN expression [41]. These results clearly indicate that miR-188-5p promotes the migration and invasion of GC cells, at least partially by suppressing PTEN.

PTEN is a well-known tumor suppressor, yet often inactivated in many types of human cancers [42, 43]. It not only involves in the regulation of apoptosis, cell cycle and angiogenesis but also plays an essential role in suppression of tumor metastasis [42, 44]. Notably, the PTEN/Akt axis regulates tumor metastasis via Wnt/β-catenin signaling [45]. Loss of PTEN function may cause the activation of Wnt/β-catenin signaling in cancers and is also associated with tumor invasion and metastasis, implying that the pro-metastatic effect of PTEN deficiency may be mediated, at least partially, by the activation of β-catenin [12]. Consistently, we also found that knockdown of *PTEN* resulted in both activation of β-catenin and promotion of cell invasion, further attesting to the concept that the β-catenin-mediated pro-invasive function due to PTEN loss might also contribute to miR-188-5p-induced metastasis in GC. In this context, our observation that PTEN-TSB significantly reversed the effect of miR-188-5p on activating Wnt/β-catenin signaling in GC cell lines, suggested that suppression of PTEN is necessary for the pro-metastatic property of miR-188-5p upregulation.

## Conclusions

In summary, our results demonstrated that miR-188-5p, by targeting PTEN, increases Akt and GSK3β phosphorylation, thus promoting β-catenin nuclear accumulation and activating Wnt/β-catenin signaling, which enhances cancer metastasis and leads to poor prognosis in patients with GC (Fig. 6d). Exploration of the function and pathogenicity of miR-188-5p not only increased our understanding of GC carcinogenesis and progression but also identified miR-188-5p as a potential molecular biomarker and therapeutic target for the diagnosis and treatment of GC.

## Additional files

Additional file 1: **Excel spreadsheet 1.** Accession codes of TCGA STAD samples. (XLSX 18 kb)

Additional file 2: **Table S1.** Clinicopathological characteristics of studied patients and expression of miR-188-5p in STAD from TCGA Dataset. (DOCX 16 kb)

Additional file 3: **Table S2.** Clinicopathological characteristics of studied patients and expression of miR-188-5p in STAD from the FMU Cohort. (DOCX 15 kb)

Additional file 4: **Table S3.** The sequences of primers used in plasmid construction. (DOCX 14 kb)

Additional file 5: **Table S4.** The sequences of primers used in real-time RT-PCR assay. (DOCX 14 kb)

Additional file 6: **Figure S1. A.** Expression of miR-188-5p and miR-188-3p in human stomach cancer clinical specimens from the TCGA miRNA HiSeq expression array data. ***, *P* < 0.001. **B.** Expression of miR-188-5p in different M (distant metastasis) classification of TCGA STAD cohort data. *, *P* < 0.05. **C.** Expression of miR-188-5p in human gastric cancer tissues and adjacent non-tumor tissues in TCGA STAD cohort data. ***, *P* < 0.001. **D.** Expression of miR-188-5p between human gastric cancer tissues and matched adjacent non-tumor tissues in TCGA STAD cohort data. Paired *t* test, *P* < 0.001. **E.** Increased fold of miR-188-5p expression between paired human gastric cancer tissues and matched adjacent non-tumor tissues samples shown in Figure S1D. (TIF 716 kb)

Additional file 7: **Figure S2.** Growth curves of the indicated cells as determined by MTT assay. Error bars represent mean ± SD from 3 independent experiments. (TIF 265 kb)

Additional file 8: **Figure S3.** qPCR analysis of miR-188-5p expression in the miR-188-5p overexpressed cells or the control cells. Error bars represent mean ± SD from 3 independent experiments. **, *P* < 0.01. (TIF 110 kb)

## Data Availability

The datasets generated and/or analysed during the current study are available from the corresponding author on reasonable request. Prediction of miR-188-5p in association with metastasis-related and Wnt/β-catenin signaling pathways was made based on the bioinformatics algorithms of gene set enrichment analysis (GSEA) through The Cancer Genome Atlas (TCGA) (http://tcga-data.nci.nih.gov/tcga).

## Abbreviations

Akt: AKT serine/threonine kinase
APC: Adenomatous polyposis coli
BMP4: Bone morphogenetic protein 4
CCK-8: The Cell Counting Kit-8
CCND1: Cyclin D1
CD44: CD44 molecule
CK1α: Casein kinase 1 alpha
CST: Cell signaling technology
DAB: Diaminobenzidine
DAPI: 4′6-diamidino-2-phenylindole
FDR: False discovery rate
FGF18: Fibroblast growth factor 18
FMU: Fujian Medical University
FZD: Frizzled
GAPDH: Glyceraldehyde-3-phosphate dehydrogenase
GC: Gastric cancer
GSEA: The gene set enrichment analysis
GSK3β: Glycogen synthase kinase 3 beta
LEF: Lymphoid enhancer binding factor
LNA: Locked nucleic acid
LRP5/6: LDL receptor-related protein 5 or 6
miR: MicroRNA
MMP7: Matrix metalloproteinase 7
MYC: MYC proto-oncogene
NC: Normal control
NGEC: Normal gastric epithelial cell
ORF: Open reading frame
OS: Overall survival
PBS: Phosphate buffer solution
PCR: Polymerase chain reaction
PI3K: Phosphatidylinositol 3-kinase
PTEN: Phosphatase and tensin homolog
qPCR: Quantitative real-time PCR
RT: Reverse transcription
SAM: The significance analysis of microarrays
Ser: Serine
SI: The staining index
STAD: Stomach adenocarcinoma
TCF: Transcription factor
TCGA: The cancer genome atlas
TSB: Target site blocker
TWIST: Twist family bHLH transcription factor
UTR: Untranslated region
