# Supplementary material for: Aberrantly expressed miR-188-5p promotes gastric cancer metastasis by activating Wnt/β-catenin signaling
Source: BMC Cancer. 2019 May 28;19:505. doi: 10.1186/s12885-019-5731-0 (PMC6537442; doi:10.1186/s12885-019-5731-0)
Supplement: Supplementary file 2 — Table S1. Clinicopathological characteristics of studied patients and expression of miR-188-5p in STAD from TCGA Dataset. (DOCX 16 kb) [file 12885_2019_5731_MOESM2_ESM.docx]

Table S1. Clinicopathological characteristics of studied patients and expression of miR-188-5p in STAD from TCGA dataset

| **Terms** | **No. of cases** | **Percentage** |
| --- | --- | --- |
| **Age (year)** |  |  |
| <60 | 120 | 31.7% |
| ≥60 | 258 | 68.3% |
| **Gender** |  |  |
| male | 249 | 65.9% |
| female | 129 | 34.1% |
| **Histological Type** |  |  |
| Stomach intestinal adenocarcinoma | 170 | 45.0% |
| Stomach adenocarcinoma | 208 | 55.0% |
| **Clinical stage** |  |  |
| I | 49 | 13.0% |
| II | 122 | 32.3% |
| III | 174 | 46.0% |
| IV | 33 | 8.7% |
| **Local invasion** |  |  |
| T1 | 20 | 5.3% |
| T2 | 75 | 19.8% |
| T3 | 180 | 47.6% |
| T4 | 103 | 27.2% |
| **Lymph node metastasis** |  |  |
| N0 | 117 | 31.0% |
| N1 | 100 | 26.5% |
| N2 | 79 | 20.9% |
| N3 | 82 | 21.7% |
| **Distant metastasis** |  |  |
| M0 | 345 | 91.3% |
| M1 | 33 | 8.7% |
| **Differentiation** |  |  |
| Grade I | 8 | 2.1% |
| Grade II | 137 | 36.2% |
| Grade III | 233 | 61.6% |
| **miR-188-5p expression** |  |  |
| miR-188-5p high | 189 | 50.0% |
| miR-188-5p low | 189 | 50.0% |
