# Supplementary material for: Aberrantly expressed miR-188-5p promotes gastric cancer metastasis by activating Wnt/β-catenin signaling
Source: BMC Cancer. 2019 May 28;19:505. doi: 10.1186/s12885-019-5731-0 (PMC6537442; doi:10.1186/s12885-019-5731-0)
Supplement: Supplementary file 3 — Table S2. Clinicopathological characteristics of studied patients and expression of miR-188-5p in STAD from the FMU Cohort. (DOCX 15 kb) [file 12885_2019_5731_MOESM3_ESM.docx]

Table S2. Clinicopathological characteristics of studied patients and expression of miR-188-5p in STAD from the FMU cohort

| **Terms** | **No. of cases** | **Percentage** |
| --- | --- | --- |
| **Age (year)** |  |  |
| <60 | 52 | 44.8% |
| ≥60 | 64 | 55.2% |
| **Gender** |  |  |
| male | 88 | 75.9% |
| female | 28 | 24.1% |
| **Clinical stage** |  |  |
| I | 23 | 19.8% |
| II | 28 | 24.1% |
| III | 47 | 40.5% |
| IV | 18 | 15.5% |
| **Local invasion** |  |  |
| T1 | 17 | 14.7% |
| T2 | 20 | 17.2% |
| T3 | 30 | 25.9% |
| T4 | 49 | 42.2% |
| **Tumor size (cm)** |  |  |
| <5 | 54 | 46.6% |
| ≥5 | 62 | 53.4% |
| **Lymph node metastasis** |  |  |
| N0 | 32 | 27.6% |
| N1 | 22 | 19.0% |
| N2 | 29 | 25.0% |
| N3 | 33 | 28.4% |
| **Distant metastasis** |  |  |
| M0 | 98 | 84.5% |
| M1 | 18 | 15.5% |
| **Differentiation** |  |  |
| Grade I | 9 | 7.8% |
| Grade II | 35 | 30.2% |
| Grade III | 72 | 62.1% |
| **miR-188-5p expression** |  |  |
| miR-188-5p high | 58 | 50.0% |
| miR-188-5p low | 58 | 50.0% |
