# Supplementary material for: Aberrantly expressed miR-188-5p promotes gastric cancer metastasis by activating Wnt/β-catenin signaling
Source: BMC Cancer. 2019 May 28;19:505. doi: 10.1186/s12885-019-5731-0 (PMC6537442; doi:10.1186/s12885-019-5731-0)
Supplement: Supplementary file 4 — Table S3. The sequences of primers used in plasmid construction. (DOCX 14 kb) [file 12885_2019_5731_MOESM4_ESM.docx]

**Table S3. The sequences of primers used in plasmid construction**

| Primers | Sequence |
| --- | --- |
| miR-188-5p | F: 5’-GAAGATCTTCAGAGCGAGCCTTCTCTTCC-3’ |
|  | R: 5’-CGGAATTCCGGGAGTCCTGAGAAGGTTCT-3’ |
| PTEN 3’UTR | F: 5’-CTAGCTAGCTAGGATATACTGGTTCACATCCTAC-3’ |
|  | R:5’-GCTCTAGAGCGATGAAGTTCTGCCTAATCT-3’ |
| PTEN ORF | F: 5’-GGGGTACCCCGCCACCATGGACTACAAGGACGATGA  TGACAAGACAGCCATCATCAAAG-3’ |
|  | R: CGGGATCCCGTCAGACTTTTGTAATTTGTG-3’ |
