# Supplementary material for: Aberrantly expressed miR-188-5p promotes gastric cancer metastasis by activating Wnt/β-catenin signaling
Source: BMC Cancer. 2019 May 28;19:505. doi: 10.1186/s12885-019-5731-0 (PMC6537442; doi:10.1186/s12885-019-5731-0)
Supplement: Supplementary file 5 — Table S4. The sequences of primers used in real-time RT-PCR assay. (DOCX 14 kb) [file 12885_2019_5731_MOESM5_ESM.docx]

**Table S4. The sequences of primers used in real-time RT-PCR assay**

| Gene Symbol | Real-time-primer-up | Real-time-primer-down |
| --- | --- | --- |
| PTEN | CGGTGTCATAATGTCTTTCAGC | TGAAGGCGTATACAGGAACAAT |
| TWIST1 | TCCATTTTCTCCTTCTCTGGAA | CCTTCTCGGTCTGGAGGAT |
| MYC | CACCGAGTCGTAGTCGAGGT | TTTCGGGTAGTGGAAAACCA |
| MMP7 | GCATCTCCTTGAGTTTGGCT | GAGCTACAGTGGGAACAGGC |
| CCND1 | GGCGGATTGGAAATGAACTT | TCCTCTCCAAAATGCCAGAG |
| CD44 | CACGTGGAATACACCTGCAA | GACAAGTTTTGGTGGCACG |
| BMP4 | GCATTCGGTTACCAGGAATC | TGAGCCTTTCCAGCAAGTTT |
| FGF18 | CTTACGGCTCACATCGTCC | ACTTCCTGCTGCTGTGCTTC |
