# Supplementary figures and images for: Aberrantly expressed miR-188-5p promotes gastric cancer metastasis by activating Wnt/β-catenin signaling
Source: BMC Cancer. 2019 May 28;19:505. doi: 10.1186/s12885-019-5731-0 (PMC6537442; doi:10.1186/s12885-019-5731-0)

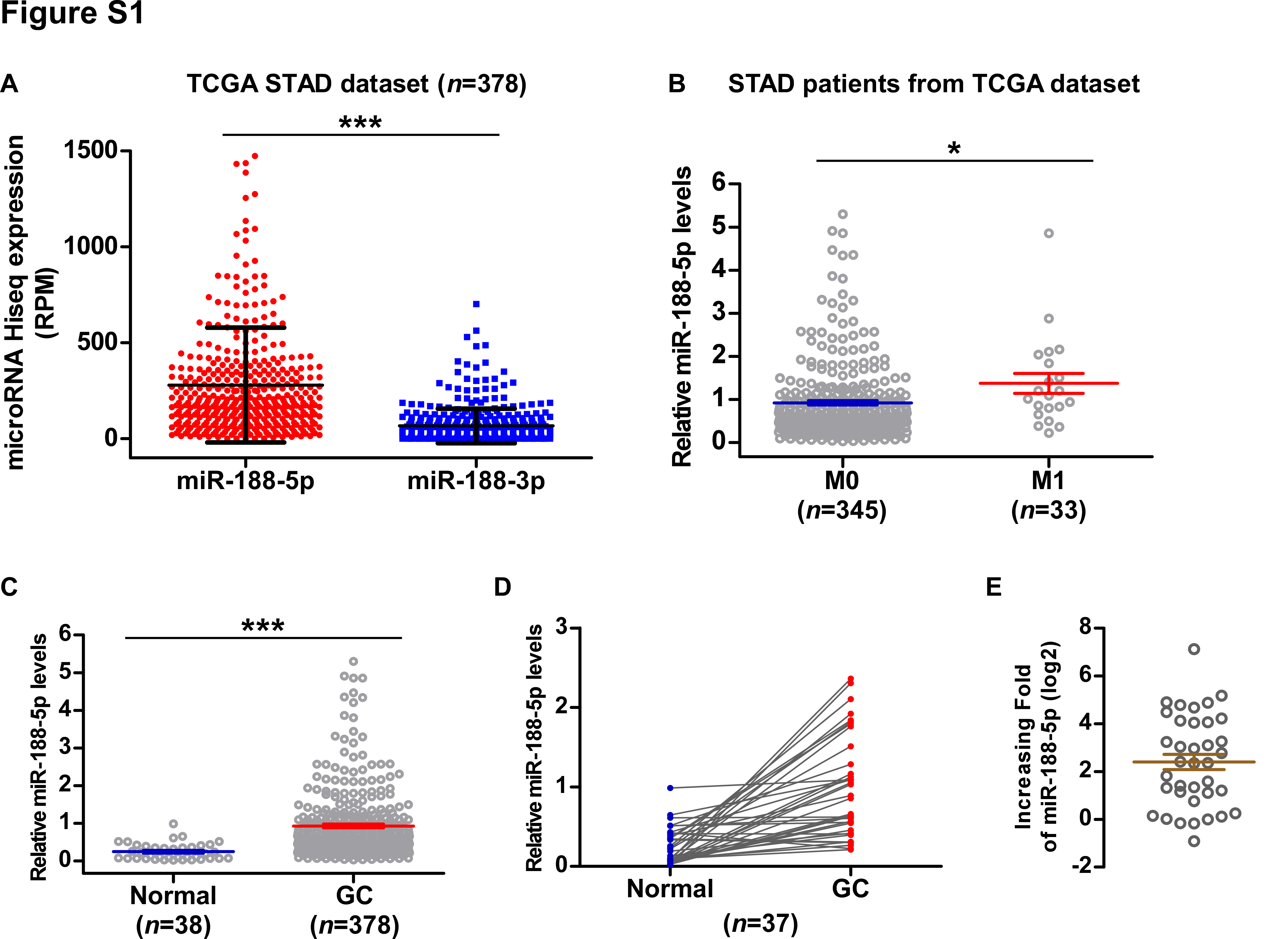

Supplement: Supplementary file 6 — Figure S1. A. Expression of miR-188-5p and miR-188-3p in human stomach cancer clinical specimens from the TCGA miRNA HiSeq expression array data. ***, P < 0.001. B. Expression of miR-188-5p in different M (distant metastasis) classification of TCGA STAD cohort data. *, P < 0.05. C. Expression of miR-188-5p in human gastric cancer tissues and adjacent non-tumor tissues in TCGA STAD cohort data. ***, P < 0.001. D. Expression of miR-188-5p between human gastric cancer tissues and matched adjacent non-tumor tissues in TCGA STAD cohort data. Paired t test, P < 0.001. E. Increased fold of miR-188-5p expression between paired human gastric cancer tissues and matched adjacent non-tumor tissues samples shown in Figure S1D. (TIF 716 kb) [file 12885_2019_5731_MOESM6_ESM.tif]

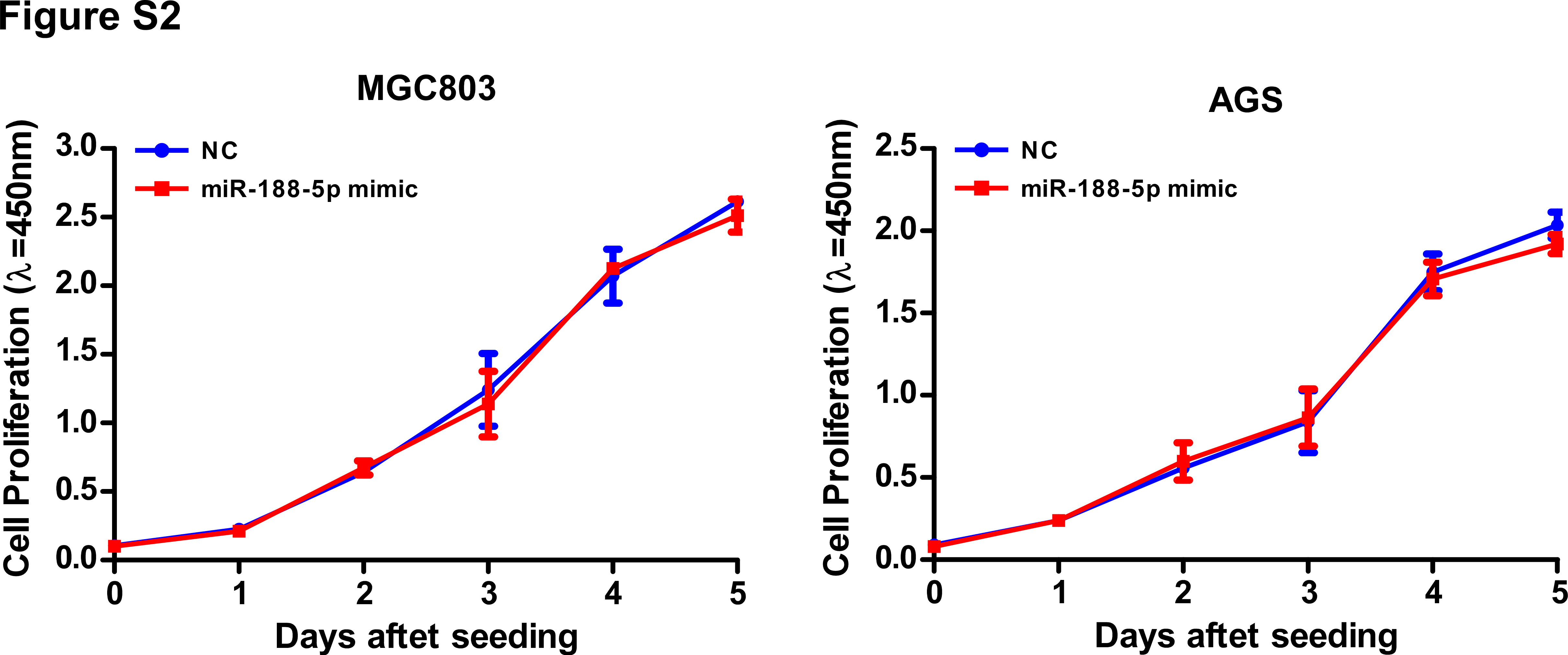

Supplement: Supplementary file 7 — Figure S2. Growth curves of the indicated cells as determined by MTT assay. Error bars represent mean ± SD from 3 independent experiments. (TIF 265 kb) [file 12885_2019_5731_MOESM7_ESM.tif]

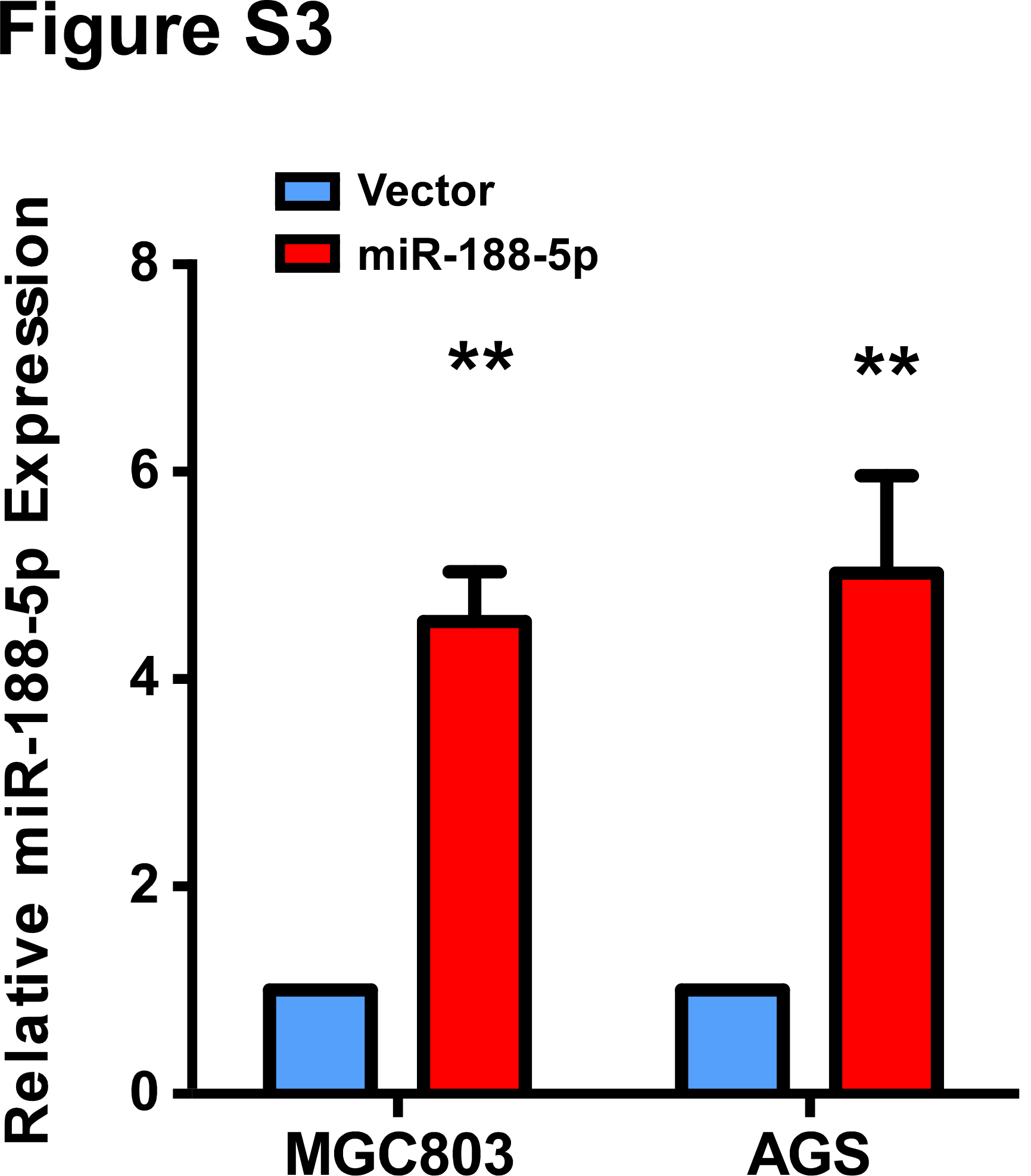

Supplement: Supplementary file 8 — Figure S3. qPCR analysis of miR-188-5p expression in the miR-188-5p overexpressed cells or the control cells. Error bars represent mean ± SD from 3 independent experiments. **, P < 0.01. (TIF 110 kb) [file 12885_2019_5731_MOESM8_ESM.tif]
